# Discussion of a Paper on “The Dentist in His Profession among the People”

**Published:** 1896-04

**Authors:** S. B. Hartman

**Affiliations:** Ft. Wayne, Ind.


### Discussion of a Paper on "The Dentist in his Profession Among the People.

BY DR. S. B. HARTMAN, FT. WAYNE, IND.

Read before the Tri-State Dental Meeting at Detroit, June, 1895, and published on page 611 in the Dental Register December last.

J. C. Walton, Norvell, Mich.: Dr. Hartman's boy, who applied to the college for admission, was a typical aspirant to dental knowledge to-day. Generally we have looked upon dentistry as a genteel pursuit, permitting support without manual toil, requiring only a clever knack of adapting oneself to circumstances, a little finger dexterity, a few dollars' worth of tools and material, and a fair share of self-assurance. Almost every one in the past did, as too many now do, neglect their preliminary education as something not needful for a successful dentist's career, while the fact is, the higher we rise in culture and refinement, the more our lives and the world must become to us. It is the small-minded men, the petty thinkers, who are finding fault with fate and quarreling with the competitors, while our great minds go on seeing great things.

Good and bad luck, too, is little else than good and bad training; it is, as a rule, quickness of mind versus ignorance. Men embrace their chances because they have education sufficiently to see them. One sees how he can supply the dental needs of a patient by doing artistic bridge-work, and pockets a large fee, another applies the forceps, and follows with a $5.00 set of artificial teeth—perception versus blindness. Not one in fifty of our fraternity are students in the sense I would have them.

The systematic use of our reason, as developed by early habits of study, must be added to mere finger-craft if we are to be considered educated dentists. We do not expect a student to make a gold crown on an incisor root unless he has exercised his mind in observing the connection of ideas, and following them in train, sees the results before he has begun, is shocked by the incongruity, and chooses a less objectionable way.

We admire the leg-motion of the dancing-master, and the finger-motion of tbe musician. The incredible and astonishing actions of the rope-dancer or tumbler are almost beyond the conception of unpracticed spectators, yet these are nothing but the mere effects of use and industry in men whose bodies have nothing peculiar in them from those of the amazed lookers-on. As it is in the body so it is in the mind. The difference so observable in men's understandings do not arise so much from natural faculties as acquired habits of use.

This, in a broad sense, is education. An educated man is one with mind trained to methodical thinking, and whose memory is a magazine of facts.

The school is the mental training ground, and books the sources of supply for his memory. It is estimated that a common school education adds 50 per cent. to the productive power of the laborer, an academic education from 200 to 300 percent.

At this to-be-memorable Tri-State Meeting, illustrations are met at every turn in the trained faculties and polished manners of the educated men we meet.

What wonder if there is lack of sympathy between such, and the "hewers of wood and drawers of water," in the profession?

Grant that we are but mechanics, and the advantages of education to mechanical skill may be illustrated by reference to Natt, Cartwright, Whitney and Fulton.

If Stephenson and Edison seem exceptions, we point you only to their hard work in self-education, and still claim that all the great steps have been taken by trained thinkers.

Dr. Hartman is also right in urging this question from the point of citizenship. Ever since our ambitious and egotistical forefathers formally declared: "We are a specialty in medicine," the obligation has been on every admirer of the code to make himself a medical man. How many have succeeded, let every one answer to his own conscience. While many have hoped to share medical honors by this flimsy feat "we are," the fact remains that before the courts and in the public mind, we are not. Then let us get all the credit we can as skilled dentists, by being what we wish to seem. Most of our enjoyment, however, will continue to come from the confidential and social positions accorded us by the citizen friends of the commonwealth in which we live.

There are evidences in our current literature that some are beginning to take statesman-like views of our profession. This is a hopeful sign. Our position relative to other professions, to our confrers, our competitions, our patients, the public, and the privileges that duty to our family dependents and ourselves demanded, seem to afford vital topics, too little discussed, that our rank, history and biography as fit subjects for dental conventions.

Dental societies have accomplished much. The representative plan of the American Dental Association and its satelites has been wise. But times are changing, and there is some evidence that the objects of organized support to the imaginary dignity of the elite. I will wait anxiously the time when dental societies shall be made attractive to the majority, and shall be the special agency for the supervision, extension and development of the profession as a whole.

Looking to the future of dentistry, the questions to-day most pertinent to its welfare are these: What are its younger members believing, and what are they doing? If it be true that the educators of to-day are making sentiment for the future, as educators of the past have influenced us. I am sorry Dr. Hartman has not considered the effect of the dentist on his profession, and among his people, who, in the interest of dental colleges, lobbys through State legislation, or who tries to place an embargo on the dentist of the poor; or who on examining boards, passes out questions he cannot answer, yet upon which applicants must be marked, or who, as college manager, plays a confidence game with innocent seekers after dental instructions, and who solicits clinical patients by the same method, and in the same spirit, which he declares unethical in individual practice.

Dr. J. Taft, Cincinnati: During the reading of the paper and the remarks of Dr. Walton, I was impressed with the thought that more attention to such subjects as this would be given by those who are teachers, and who have influence on those who are coming into the profession in the future. We should endeavor to stimulate young men on this line. It is a good thing for them to appreciate that there is something more to be done by the dentist than the mere hum-drum work of his office. The broader his intelligence, the more power he will exercise over his fellow-men. We find that illustrated in the medical profession. I fear that our educational institutions are not exercising all the influence they should in this direction of stimulating young men to broader views than they possess when they come from the colleges. Occasionally we find a young man well educated, and he goes out under vastly more suspicious circumstances than the young man who is crowded into the narrow groove of mere routine professional work. Though he may understand all the branches of the curriculum so that as he is able to pass them, there is still a broader range of knowledge that he ought to have, and that would help him greatly in his special work. This range includes the progress that is being made in the various departments of science, theoretical and applied. Electricity is an illustration of this, and hundreds of other things illustrate it as well. Of course no one man can understand everything. But it is well that there should be a broad view of all these general subjects of the times so far as possible. Last night we had an interesting presentation of the subject of bacteriology by Dr. Barrett. How many of us understand that subject as we ought? So we should have more knowledge of all the range of subjects that come within our purview, and then we shall better understand the causes of things, and do better work, than if we are simply crowded down to the narrow space of the every day technical and operative duties of our profession.

W. C. Barrett: This subject touches me at a very tender point. The influence that each one exerts cannot be estimated. The key-note that is set at the commencement of a dental meeting will vibrate through every session. The influence of leading men, like Chapin A. Harris, like Bond, and others to whom I might refer, not to speak of some of our venerable friends here present, has always been a powerful agent for the elevation of the profession. It is the duty of every dentist to feel that he is living in the eyes of the world; that he is the representative of a formative profession. At this time it is blossoming out, the calyx is opening, and we live in the hope that hereafter the fruit shall ripen for the harvest. Dentists sometimes forget this. We are not living here simply for the amount of money we can make. The man who devotes himself entirely to money-grubbing is not the man who gets the most out of life. My life has not been an unhappy one, though I cannot say it has been particularly a success. The reason why I have been measurably happy, is because I have been a hard working man, have been busy every day; I can get my recreation out of my labor. The days frequently are not long enough for me. My heart is in my work, and I am determined it shall remain there, and hence I can get some enjoyment out of it.

There is not an individual so humble that he does not attune some other heart. The violin lay upon the piano, across which the master's hand was sweeping. I took up the violin to remove it, and found it throbbing, pulsating, vibrating in my hand like a thing of life; all resonant with the harmony derived from its contact with the piano. So the very contact with an active mind makes our own hearts sing with joy, makes them vibrate with all the activities of life. And when we put our minds in concord with others, when our intellects are attuned to the same key-note, when our hearts vibrate in harmony with them, how much we can do for the benefit of our fellows.

JOSEPH LATHROP, of Detroit, read a poem, entitled "Fraternal Love," written by Dr. J. A. Robinson, a practitioner of sixty years, the poem being delicated to the Tri-State Meeting.

